# The Correction of Injury Resulting from Extraction

**Published:** 1895-02

**Authors:** S. E. Davenport


### THE CORRECTION OF INJURY RESULTING FROM EXTRACTION.[1]

BY S. E. DAVENPORT, D.D.S., M.D.S.

WHILE this is not intended specially as a plea for conservative practice, it serves as a fitting opportunity for the condemnation of inflexible rules governing either the choice of filling-materials or methods for the correction of irregularities, instead of basing the decision in every case upon existing circumstances and conditions.

The writer will never forget the horror with which he heard a prominent dentist give as his advice for the correction of three overcrowded mouths, the casts being before him and differing much in typical characteristics, that the sixth-year molars in all three cases should be extracted, adding that he saw but few crowded arches which similar treatment would not correct.

That was at least ten years ago, long before Dr. I. B. Davenport's comprehensive explanation of the ideal and warning to all practitioners against a disturbance of the same, which studious essay has, in the writer's opinion, done as much to establish the habit of original thinking and of saving teeth from the forceps as any paper of recent years.

Any single illustration of harm resulting from the extraction of sixth-year molars will not, as is well understood, serve to condemn the method in its entirety, but as the case which is to be presented this evening received the combined advice of four well-known dentists, three of whom are members of the New York Odontological Society, it will at least prove that the conditions which seemed to those gentlemen a proper basis for such advice were not principles, but prejudices.

In 1882 the patient, then a resident of Brooklyn, a boy of thirteen years, was taken by his parents to their dentist, who advised the extraction of the sixth-year molars, hoping thereby to correct a slight prominence of the lower incisors, and what seemed to be a

---

[1] Read before the New York Odontological Society, October 16, 1894.

too crowded condition of the teeth, both upper and lower. He was encouraged, perhaps, to give the opinion by the presence of compound cavities, anterior-proximal and crown, in the lower molars.

The parents were not pleased with the advice, and, during the following eighteen months, consulted two other Brooklyn dentists, and, finally, at the suggestion of their family dentist, brought the boy to New York, to get the advice of one of his prominent friends here.

As above stated, all four dentists agreed, and late in 1883, when the youth was fourteen years old, the four teeth in question were removed.

Fortunately for the evidence the teeth were preserved, and have since been seen by the writer, the upper ones having only small crown cavities, and while the decay in the lower teeth was extensive, there is no question but that any one of the four gentlemen could have so filled them that the teeth would have been preserved a lifetime.

The decay in all four teeth must certainly have been much less when their extraction was first advised than it was eighteen months later, when they were removed, and the force, therefore, of existing decay as a corroborative reason for such advice was much less at the earlier period, and was entirely insufficient even at the time of extraction.

When the patient came into the writer's hands five years later, almost the first question the mother asked was, "Why is it that my boy will persist in masticating almost entirely with his front teeth, in spite of my calling his attention to so bad a habit almost every day?"

The boy had actually no points of contact other than the occlusion of the wisdom-teeth and of the central and lateral incisors.

It may be interesting also to state that about this time the youth was suffering from so-called nervous dyspepsia, and was receiving treatment for it at the hands of a well-known specialist.

At this point of the story it seems fitting to appeal to all who have the advancement of their profession at heart not to advise the extraction of teeth for the purpose of regulation without first taking casts, so that the relations existing between the lingual and palatal surfaces of the teeth may be observed.

Frequently this occlusion of the inner surfaces has an important bearing upon the opinion formed and the advice given, and the casts serve as a most important basis for the intelligent study later, when the results of such extraction are accomplished.

Children are sometimes counselled by their elders to count twenty when tempted to make an angry retort, expecting in that way to cause one to modify his answer; and as, in the opinion of the writer, by far too many teeth are still sacrificed with the fallacious idea that room which can be utilized is being gained, the rule to always take casts and study them before advising extraction would not only serve to teach the dentist many principles, but through his greater knowledge of nature's laws would cause him to be less frequently a murderer of the dental organs.

As the wisdom-teeth were fully erupted when the writer first saw this young gentleman, there was no hope that either growth or development would ever cause such occlusion of the bicuspids and twelfth-year molars as would enable him to properly masticate.

After expanding the upper arch slightly in the twelfth-year molar and second bicuspid region for the purpose of bringing about a better relation between the upper and lower teeth, it was decided to build up largely with gold the lower twelfth-year molars, and to build down the four upper bicuspids and the upper twelfth-year molars.

While it was not possible to give an ideal masticating surface according to Dr. I. B. Davenport's illustration of planes and angles, the result was so satisfactory that almost immediately the habit of masticating with the front teeth became a thing of the past, and the remedies administered by the noted specialist became so efficacious that the dyspepsia was cured.

At the time this case was accepted an effort was made to secure casts of the mouth as it was before the teeth were extracted, but none of the gentlemen whose advice was followed had taken casts, all having given their opinions condemning the four large teeth, after a hasty and necessarily superficial examination.

A glance at the method adopted for hinging the casts which are being passed around, so that the inner surfaces of the teeth can be studied when the jaws are closed, will explain the assertion made above, that any examination of the natural teeth alone must be, necessarily, too superficial to serve as a basis for the condemnation of teeth.

If any present are inclined to doubt that the loss of the sixth-year molars in this mouth was the cause of the deplorable condition afterwards found there, they are referred to the cast first shown, where the characteristic tipping of the teeth will, the writer thinks, prove his position to be correct.

